# Corticolimbic Expression of TRPC4 and TRPC5 Channels in the Rodent Brain

**DOI:** 10.1371/journal.pone.0000573

**Published:** 2007-06-27

**Authors:** Melissa A. Fowler, Kyriaki Sidiropoulou, Emin D. Ozkan, Christopher W. Phillips, Donald C. Cooper

**Affiliations:** 1 Psychiatry Department, University of Texas Southwestern Medical Center, Dallas, Texas, United States of America; 2 Computational Biology Lab, Institute of Molecular Biology and Biotechnology (IMBB)-Foundation for Research and Technology (FORTH), Vassilika Vouton, Heraklio, Greece; Columbia University, United States of America

## Abstract

The canonical transient receptor potential (TRPC) channels are a family of non-selective cation channels that are activated by increases in intracellular Ca^2+^ and G_q_/phospholipase C-coupled receptors. We used quantitative real-time PCR, in situ hybridization, immunoblots and patch-clamp recording from several brain regions to examine the expression of the predominant TRPC channels in the rodent brain. Quantitative real-time PCR of the seven TRPC channels in the rodent brain revealed that TRPC4 and TRPC5 channels were the predominant TRPC subtypes in the adult rat brain. In situ hybridization histochemistry and immunoblotting further resolved a dense corticolimbic expression of the TRPC4 and TRPC5 channels. Total protein expression of HIP TRPC4 and 5 proteins increased throughout development and peaked late in adulthood (6–9 weeks). In adults, TRPC4 expression was high throughout the frontal cortex, lateral septum (LS), pyramidal cell layer of the hippocampus (HIP), dentate gyrus (DG), and ventral subiculum (vSUB). TRPC5 was highly expressed in the frontal cortex, pyramidal cell layer of the HIP, DG, and hypothalamus. Detailed examination of frontal cortical layer mRNA expression indicated TRPC4 mRNA is distributed throughout layers 2–6 of the prefrontal cortex (PFC), motor cortex (MCx), and somatosensory cortex (SCx). TRPC5 mRNA expression was concentrated specifically in the deep layers 5/6 and superficial layers 2/3 of the PFC and anterior cingulate. Patch-clamp recording indicated a strong metabotropic glutamate-activated cation current-mediated depolarization that was dependent on intracellular Ca^2+^and inhibited by protein kinase C in brain regions associated with dense TRPC4 or 5 expression and absent in regions lacking TRPC4 and 5 expression. Overall, the dense corticolimbic expression pattern suggests that these Gq/PLC coupled nonselective cation channels may be involved in learning, memory, and goal-directed behaviors.

## Introduction

The dynamic homeostatic mechanisms that neurons use to regulate intracellular Ca^2+^ signaling have received much attention recently, due to the important role Ca^2+^ plays in cellular processes including gene expression, axon growth, synaptic plasticity and cell death. TRPC channels have been identified as important channels that may be involved in maintaining intracellular Ca^2+^ concentrations in response to a range of signaling modalities [1]. Despite the recent interest and potential importance of the TRPC channels, there have been no thorough descriptions of the expression pattern of these channels in the mammalian brain.

**Figure 1:**
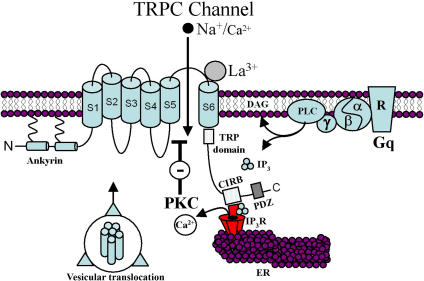
TRPC channel structure and mechanisms of activation. Schematic showing the six transmembrane structure of the nonselective cation channels, TRPC4 and 5, and the conserved N-terminal ankryin and C-terminal TRP, CIRB, and PDZ domains. The channels are proposed to be activated by releases of intracellular Ca^2+^ stores from the ER, conformational changes following binding of IP_3_ to IP_3_R, and vesicular translocation. The channels are inhibited by PKC and potentiated by La^3+^.

**Figure 2:**
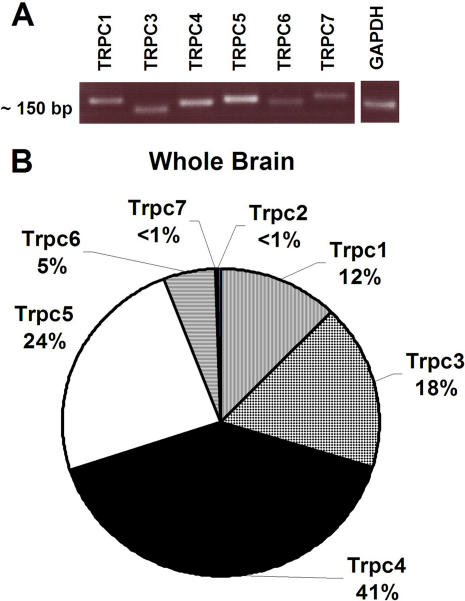
Expression of the TRPC channels in the rat brain. (A) Agarose gel showing a single product from real-time PCR of the TRPC channels (B) Pie chart showing the relative expression of the seven TRPC channels in rat whole brain determined using real-time PCR. Results was normalized to GAPDH mRNA levels. (n = 4; TRPC1: Avg = 0.0012, SEM = ±0.00011; TRPC3: Avg = 0.0018, SEM = ±0.00015; TRPC4: Avg = 0.0046, SEM = ±0.00037; TRPC5: Avg = 0.0022, SEM = ±0.000356; TRPC6: Avg = 0.00055, SEM = ±0.000055; TRPC7: Avg = 0.000027, SEM = ±0.0000054)

The TRPC non-selective cation channels consist of seven members that are organized into four groupings based on sequence homology and functional similarities: TRPC1, TRPC2, TRPC3/6/7, and TRPC4/5 [2],[3]. These channels are mixed cation (K^+^, Na^+^ and Ca^2+^) channels that are activated by Gq-coupled receptors, such as group 1 mGluR and muscarinic acetylcholine receptors [4],[5],[2],[6],[7],[8]. To date, there are no selective drugs capable of distinguishing between the TRPC subtypes. However, micromolar concentrations of the trivalent lanthanoids (La^3+^, Gd^3+^) block TRPC3/6/7 but potentiate TRPC4/5 channels [9]. In cell culture expression systems TRPC4 and 5 channels function to modulate cellular excitability, neuronal growth and axon guidance, and regulation of Ca^2+^ homeostasis [4], [2], [10],[11]. The TRPC4 and 5 channels have been proposed to be activated by Gq/phospholipase C signaling, release of intracellular Ca^2+^ stores, or vesicular translocation to the membrane (Figure 1; [2], [12], [10]). Gq-signaling-mediated activation of phospholipase C increases inositol triphosphate (IP_3_) that binds to the IP_3_ receptor located on the endoplasmic reticulum and releases intracellular Ca^2+^ [13]. A conformational change opens the TRPC channel bound to IP_3_ receptors, which bind to the C-terminal end of the TRPC channels via the calmodulin/IP_3_ receptor binding domain [12],[14].

Another mechanism of activation for TRPC5 channels is vesicular translocation to the membrane following stimulation by neuronal growth factors [15],[10]. TRPC5 is expressed in neuron growth cones and is rapidly inserted into the membrane following stimulation by neuronal growth factors where it leads to cessation of growth in cultured cells [15],[10]. These functional in vitro studies suggest that TRPC5 channels may be expressed presynaptically in axon terminals and involved in axon guidance [16].

Although a few reported studies have examined TRPC channels in select brain regions, an extensive and comprehensive description of the brain-wide expression of TRPC4 and 5 channels is lacking [17],[18],[19],[20],[21],[22],[23]. In this study we sought to identify the expression pattern and suggest a possible function of the TRPC4 and 5 channels in the rodent brain.

## Results

### Quantitative real-time PCR expression of the seven TRPC channels in rat brain

Results of real-time PCR performed on RNA isolated from adult rat whole brain (excluding the cerebellum) show that TRPC4 and 5 are the two predominantly expressed TRPC channels in the brain, comprising approximately 41% and 24% of the TRPC channel population respectively (Figure 2). TRPC3, TRPC1, and TRPC6 are moderately expressed at 18%, 12%, and 5% of the population respectively. TRPC2 and TRPC7 show very little expression in the brain at less than 1%. The real-time PCR primers were designed to target all known splice variants of the TRPC channels and produce a single product (Table 1). The binding efficiency for each primer set was calculated in order to account for any differences in primer binding and the results were normalized to the housekeeping gene, GAPDH, in order to compare the expression of the different TRPC channels.

**Table 1:** Real-time PCR primer sequences for the seven TRPC channels in rat

Gene	Acc. #	Forward primer	Reverse primer
**TRPC1**	NM 053558	5′AGGTGAAGGAGGAGAACACCTTG 3′	5′CCATAAGTTTCTGACAACCGTAGTCC 3′
**TRPC2**	NM 022638	5′AGAAGCTGGGCAATTTCAACG 3′	5′CGATGAGCATGTTGAGTAGCACA 3′
**TRPC3**	NM 021771	5′CCACATGCAGTGAGACTTTGACTC 3′	5′AGGCCAACCTTGGGATCATTT 3′
**TRPC4**	NM 053434	5′AATTACTCGTCAACAGGCGGC 3′	5′CACCACCACCTTCTCCGACTT 3′
**TRPC5**	NM 080898	5′AAGTTTCGAATTTGAGGAGCAGATG 3′	5′AATCTCTGATGGCATCGCACA 3′
**TRPC6**	NM 053559	5′GCCTCATGATTATTTCTGCAAGTGTAC 3′	5′TGAACTCTTTCTCAATGTTGGCAA 3′
**TRPC7**	XM 225159	5′ATGACGAGTTCTATGCCTACGACG 3′	5′TTGTAGGCATTCATACGGGAGC 3′

Table showing the left and right real-time PCR primer sequences for the seven TRPC channels in rat and GAPDH and the NCBI accession numbers for each gene.

### Distribution of TRPC4 and 5 in the rodent brain

**Figure 3:**
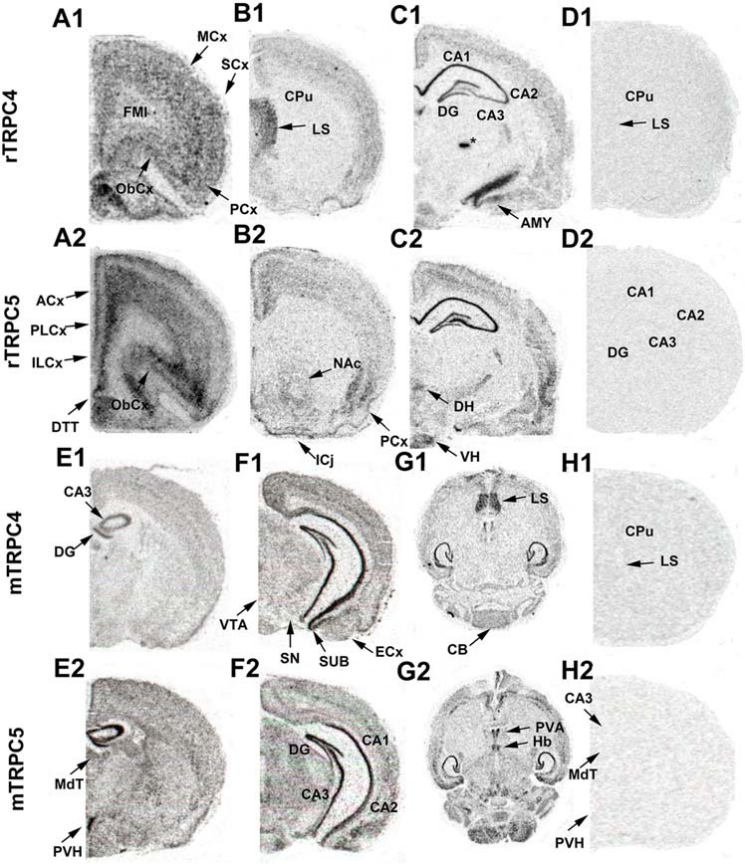
Expression of TRPC4 and 5 in rat and mouse brain. (A1, B1, C1) In situ hybridization of TRPC4 in rat coronal brain slices. (Bregma, mm): A1, 3.70; B1, 1.00; C1, −4.16 (A2, B2, C1) In situ hybridization of TRPC5 in rat coronal brain slices. Coordinates same as A1,B1,C1. (D1, H1) Unlabeled S^35^-labeled probe control in situ hybridization of TRPC4 and TRPC5 in rat coronal brain slices. (E1, F1) In situ hybridization of TRPC4 in mouse coronal brain slices. (G1) In situ hybridization of TRPC4 in horizontal mouse brain slices. (E2,F2) In situ hybridization of TRPC5 in mouse coronal brain slices. (G2) In situ hybridization of TRPC5 in mouse horizontal brain slices. (D2, H2) Unlabeled S^35^-labeled probe control in situ hybridization of TRPC4 and TRPC5 in mouse coronal brain slices.

The qualitative expression of TRPC4 and 5 mRNA throughout the rat and mouse brain is presented in Table 2. The results of in situ hybridization of TRPC4 were observed to be similar between the species and are therefore shown together. Coronal and horizontal brain slices show that TRPC4 mRNA is highly expressed in the LS and cell body layer of the CA1-CA2 sub-regions of the HIP, vSUB, and dorsal tenia tecta (Figure 3). Moderate expression was observed in the frontal cortex including the PFC (infralimbic and prelimbic), anterior cingulate, MCx, SCx, entorhinal cortex, piriform cortex, orbitofrontal cortex, amygdala, ventral hypothalamus, Purkinje and granule cell layers of the cerebellum. No specific labeling was detected in slices that were co- incubated with unlabeled control in situ hybridization probes for TRPC4 and 5. (Figure 3)

**Table 2:** Qualitative analysis of TRPC4 and TRPC5 mRNA expression in the rodent brain

	TRPC4	TRPC5		TRPC4	TRPC5
**Amygdala**	**++**	**+**	**Motor cortex**		
**Caudate putamen**	**0**	**0**	** Layer 1**	**0**	**0**
**Cerebellum**			** Layer 2/3**	**++**	**++**
** Molecular layer**	**0**	**+**	** Layer 5/6**	**++**	**++**
** Purkinje cell layer**	**++**	**++**	**Nucleus accumbens**		
** Granule cell layer**	**++**	**++**	** Core**	**0**	**+**
**Corpus collosum**	**0**	**0**	** Shell**	**0**	**++**
**Entorhinal cortex**	**++**	**+++**	**Orbitofrontal cortex**	**++**	**+++**
**Habenula**	**++**	**+++**	**Piriform cortex**	**++**	**+++**
**Hippocampus**			**Prefrontal cortex**		
** CA1**	**+++**	**+++**	** Layer 1**	**0**	**0**
** CA2**	**+++**	**+++**	** Layer 2/3**	**++**	**+++**
** CA3**	**++**	**+++**	** Layer 5/6**	**++**	**+++**
** Hilus**	**+**	**+++**	**Somatosensory cortex**		
** Dentate gyrus**	**++**	**++**	** Layer 1**	**0**	**0**
**Hypothalamus**			** Layer 2/3**	**++**	**++**
** Dorsal**	**0**	**+**	** Layer 5/6**	**++**	**++**
** Ventral**	**++**	**++**	**Subiculum**	**+++**	**++**
** Paraventricular**	**+**	**+++**	**Substantia nigra**	**+**	**0**
**Islands of Caleja**	**0**	**+++**	**Tenia tecta**	**+++**	**++**
**Lateral septum**	**+++**	**0**	**Thalamus**	**0**	**++**
**Medial Septum**	**+**	**0**	**Rank 0<+<++<+++**		

Qualitative analysis of the in situ hybridizations of TRPC4 and TRPC5 mRNA in rat and mouse coronal brain sections (from Figure 3). (0) no expression, (+) little expression (++) moderate expression, (+++) high expression. (n = 3)

**Figure 4:**
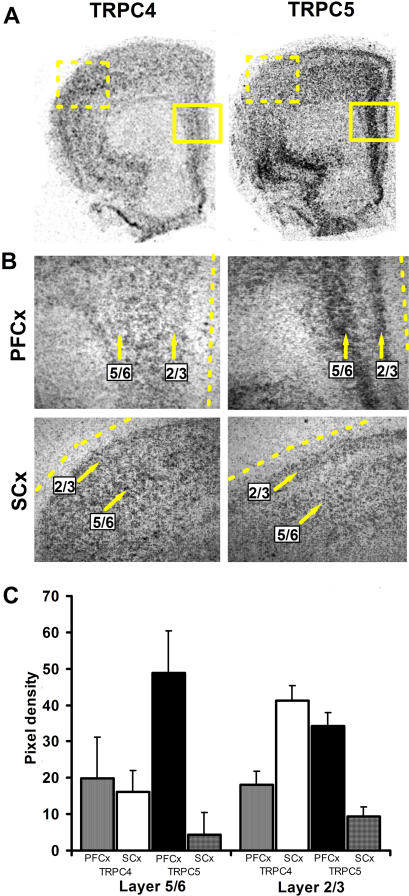
Quantification of the cRNA labelling densities of TRPC4 and 5 in the cortical layers of rat. (A) (Left) In situ hybridization of TRPC4 in a rat coronal brain slice. (Right) In situ hybridization of TRPC5 in a rat coronal brain slice. Scale bars in µm. (B) (Upper) Magnification of TRPC4 5 cRNA labeling in the PFC layers. (Lower) Magnification of TRPC4 5 cRNA labeling in the SCx layers. (C) Quantification of the cRNA labelling densities of TRPC4 and 5 in the PFC and SCx layers expressed as a % change from background (n = 3). Scale bars in µm.

To examine the cortical layer-specific expression of TRPC4 and 5 in the frontal cortex, we performed quantitative analysis of the in situ hybridization signal in the superficial (layers 2/3) and deep (layer 5/6) cortical layers. Results show that TRPC4 mRNA is expressed at similar levels throughout layers 2,3,5, and 6 in all regions of the frontal cortex, including the PFC, anterior cingulate, MCx, SCx and orbitofrontal cortex. In layers lacking pyramidal neurons, such as layer 1 of the frontal cortex, there is no TRPC4 mRNA expression (Figure 4).

**Figure 5:**
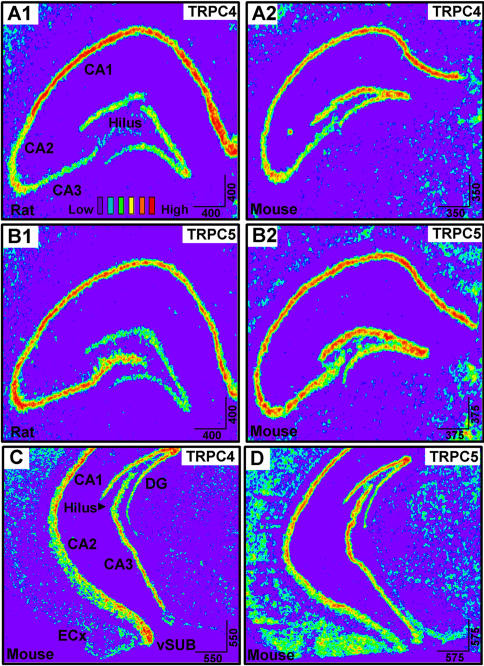
Expression of TRPC4 and TRPC5 in the hippocampal formation. (A1, A2) cRNA labelling of TRPC4 mRNA in rat and mouse CA1-3, hilus, and dentate gyrus (B1, B2) cRNA labelling of TRPC5 mRNA in rat and mouse CA1-3, hilus, and dentate gyrus (C) cRNA labelling of TRPC4 and 5 in the ventral mouse hippocampal formation including the entorhinal cortex and ventral subiculum.

**Figure 6:**
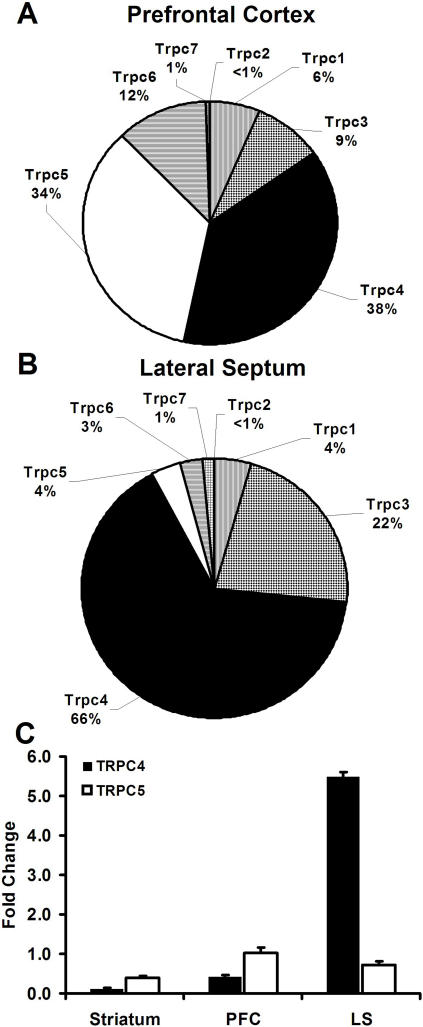
Region-specific expression of TRPC4 and TRPC5 mRNA. (A–B) Pie charts showing the relative expression of the seven TRPC channels in rat prefrontal cortex (A) and lateral septum (B). Results was normalized to GAPDH mRNA levels. (n = 4; PFC TRPC1: Avg = 0.00055, SEM = ±0.000035; TRPC3: Avg = 0.00073, SEM = ±0.000066; TRPC4: Avg = 0.0031, SEM = ±0.00035; TRPC5 Avg = 0.0021, SEM = ±0.0010; TRPC6: Avg = 0.00010, SEM = ±0.000038; TRPC7: 0.000049, SEM = ±0.0000021; LS TRPC1: Avg = 0.0011, SEM = ±0.000020; TRPC3: Avg = 0.000082, SEM = ±0.000042; TRPC4: Avg = 0.0051, SEM = ±0.000011; TRPC5: Avg = 0.0015, SEM = ±0.00015; TRPC6: Avg = 0.00016, SEM = ±0.0000063; TRPC7: Avg = 0.00015, SEM = ±0.0000014) (C) Quantification of TRPC4 and TRPC5 mRNA levels in the striatum, prefrontal cortex (PFC), and lateral septum of adult rat using real-time PCR. Results was normalized to GAPDH mRNA levels. (n = 4)

Qualitative comparisons of TRPC4 mRNA expression in the cell body layer of the dorsal HIP of adult rat and mouse were similar. In both species TRPC4 expression is very high in CA1 while moderate expression in CA2 and DG, and little expression in CA3 and hilus is observed. Analysis of ventral HIP showed that TRPC4 is highly expressed in the vSUB with less expression in the entorhinal cortex (Figure 5). In subcortical structures, there is moderate expression of TRPC4 mRNA in the amygdala, and ventral hypothalamus. In contrast, very low expression of TRPC4 mRNA is seen in either the dorsal striatum or nucleus accumbens, which matches the real-time PCR data indicating low expression of TRPC4 and 5 mRNA in the striatum relative to whole brain TRPC4 and 5 levels (Figure 6).

TRPC5 mRNA in situ hybridization indicates high expression concentrated in the deep layers 5/6 and superficial layers 2/3 of the PFC, vSUB, piriform, orbitofrontal, and entorhinal cortices (Figure 4). As with TRPC4, no significant TRPC5 expression was detected in layer 1 of the PFC where there are few neuronal cell bodies. TRPC5 had little mRNA expression in the MCx or SCx. The cell body layer of the HIP had robust expression of TRPC5 in CA1, CA2, CA3, and hilus, and moderate expression in the DG and the vSUB (Figure 5).

In subcortical structures high TRPC5 mRNA expression was detected in the Islands of Caleja, paraventricular hypothalamus, and dorsal and ventral hypothalamus. Little expression of TRPC5 is seen in the MCx, SCx and no expression is seen in the dorsal striatum although, interestingly, moderate expression of TRPC5 was detected in the nucleus accumbens (Figure 3, Table 2).

### Relative TRPC channel expression in the prefrontal cortex and lateral septum

In situ hybridization results indicate that TRPC4 or 5 mRNA is highly expressed in the LS and PFC. To validate this using real-time PCR, we examined the relative expression of all TRPC channels in the PFC and LS of age matched adult rat. PCR of the seven TRPC channels in microdissections of the PFC indicate that TRPC4 and 5 mRNAs are the most highly expressed TRPC channels in PFC, representing 38% (TRPC4) and 34% (TRPC5) of the TRPC channel population. TRPC6, TRPC3, and TRPC1 had low mRNA expression in the PFC with 12%, 9%, and 6% respectively. TRPC7 and TRPC2 comprise <1% of the TRPC channel population in the PFC (Figure 6).

Real-time PCR of the seven TRPC channels in microdissections of the LS of adult rat confirms that TRPC4 is the predominantly expressed channel in the LS, comprising 66% of the TRPC channel population. TRPC3 is moderately expressed in the LS with 22%, and TRPC5, TRPC6, and TRPC1 have lower expression with 4%, 4%, and 3% respectively. TRPC2 and TRPC7 comprise less than 1% of the TRPC channel population in the LS (Figure 6).

### Region-specific protein expression of TRPC4 and TRPC5

**Figure 7:**
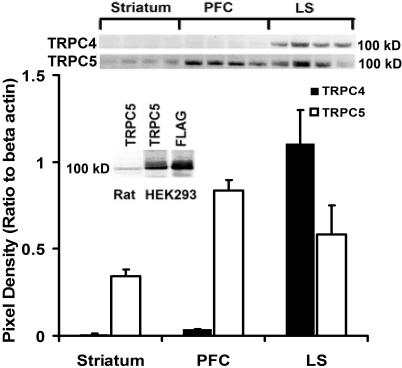
Region-specific expression of TRPC4 and TRPC5 protein. Graph shows the quantification of TRPC4 and TRPC5 protein levels in the striatum, prefrontal cortex, and lateral septum in the adult rat. Results was normalized to β-actin protein levels (Top) Representative bands of the immunoblots (n = 4). (Left inset) Immunoblot of rat brain tissue lysates and TRPC5 expressing HEK293 cells blotted with α-TRPC5 and of TRPC5 expressing HEK293 cells blotted with α-Flag.

To examine whether the mRNA expression of TRPC4 and TRPC5 corresponds with the protein expression of these channels, we performed immunoblots for TRPC4 and TRPC5 protein in microdissections from striatum, PFC, and LS. We did not examine the relative protein expression of the TRPC4 and 5 in the brain due to potential differences in antibody affinities between the two channels. Due to these inherent limitations in immunoblotting, we restricted our protein analysis to a region-specific analysis [24]. The antibody used for TRPC4 has previously been validated and shown to be specific [25]. To verify the specificity of the TRPC5 commercial antibody we cloned TRPC5 into the mammalian transfection vector, pcDNA3.1-FLAG that inserts a FLAG tag on the C-terminal region of the protein. Immunoblots of protein harvested from HEK293 cells 48 hours after transfection with TRPC5-FLAG were blotted with both α-FLAG and α–TRPC5 antibodies. The detected bands were identical in size (inset, Figure 7), confirming that the FLAG-tagged protein expressed in the HEK293 cells is TRPC5 protein. No bands were detected on immunoblots of protein harvested from cells transfected with empty vector or from non-transfected cells (data not shown). In addition, immunoblots of protein harvested from TRPC5-FLAG transfected HEK293 cells and rat whole brain show identical bands, thus confirming the specificity of the TRPC5 antibody in rat brain lysates (inset, Figure 7).

Results of TRPC5 protein expression from immunoblots from rat striatum, PFC, and LS show that TRPC4 protein is most highly expressed in the LS and moderately expressed in the PFC with lower expression in the striatum. TRPC5 protein is most highly expressed in the PFC and moderately expressed in the LS with lower expression in the striatum (Figure 7). These results show that in general the protein expression of these channels match the mRNA expression levels in these regions.

### Developmental expression of TRPC4 and 5 proteins

**Figure 8:**
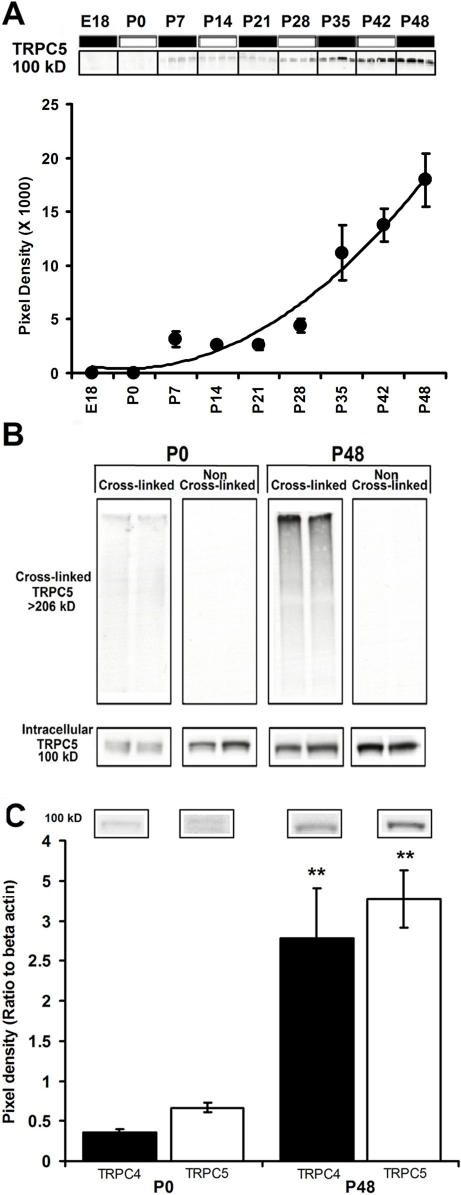
Developmental and surface expression of TRPC5 protein in the mouse hippocampus. (A) Quantification of TRPC5 protein levels in the HIP of E18 through P48 day old mice. (n = 4) (Top inset) Representative bands from the immunoblot showing TRPC5 expression in the HIP of E18-P48 mice. (B) Representative bands from an immunoblots showing surface (cross-linked) and intracellular TRPC5 protein in P0 and P48 mice (n = 6). (C) Quantification of TRPC4 and 5 protein levels in the HIP of P0 and P48 mice (n = 6; for P0, p = 0.002; for P48, p = 3.16e-5) (top inset) Representative bands from an immunoblots showing TRPC4 and TRPC5 protein in the HIP of P0 and P48 mice (n = 6)

**Figure 9:**
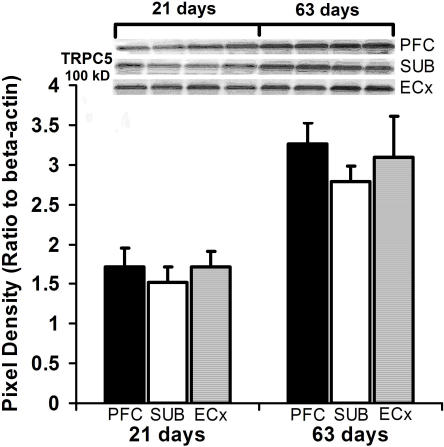
Developmental expression of TRPC5 protein in the rat brain. Quantification of TRPC5 protein levels in the prefrontal cortex (PFC), subiculum (SUB), and entorhinal cortex (ECx) in 21 and 63 day old rats. Results were normalized to β-actin protein levels. (Top left Inset) Representative bands of the immunoblot (n = 4)

To determine if TRPC4 or 5 protein expression is developmentally regulated, we examined expression at different stages of development in the HIP. Immunoblots performed for TRPC4 and 5 protein in microdissections of HIP from post-natal day 0 (P0) and post-natal day 48 (P48) mice show that TRPC4 and 5 protein levels are higher at P48 compared to P0 (Figure 8C). HIP TRPC5 protein is present at low levels from embryonic day 18 (E18) E18 to P20 and increase robustly from P20 to P48 (Figure 8B). Similar results were observed in rat PFC, vSUB, and ECx comparing post-natal day 21 (P21) and post-natal day 63 (P63) rats (Figure 9).

To examine the surface expression of TRPC5 protein in P0 and P48 day old mice, we used the irreversible, membrane impermeable crosslinker, BS^3^ to cross-link surface-expressed TRPC proteins. Cross-linked samples were blotted and the ∼100 kD band representing the remaining intracellular pool of TRPC5 was quantified. These methods are similar to those described by Grosshans et al [26]. Cross-linking resulted in immunoblots that showed a TRPC5 labeled high molecular weight smear (>206 kD) in cross-linked samples that was not present in non-cross-linked samples, indicating successful surface cross-linking (Figure 8B). This procedure produces a reduction in the TRPC5 ∼100 kD band in cross-linked samples compared to non cross-linked sample which reflects TRPC5 surface expression. (Figure 8B). To ensure that cross-linking produces no non-specific changes in protein we examined levels of the intracellular protein, beta-actin, and found that the total amount of protein was not significantly different across cross-linked and non cross-linked samples for P0 or P48 (Figure S1). This confirms previous work demonstrating the lack of membrane permeability of BS^3^ [27].

### Expression of TRPC4 and TRPC5 channels is associated with a burst-induced delayed after-depolarization

**Figure 10:**
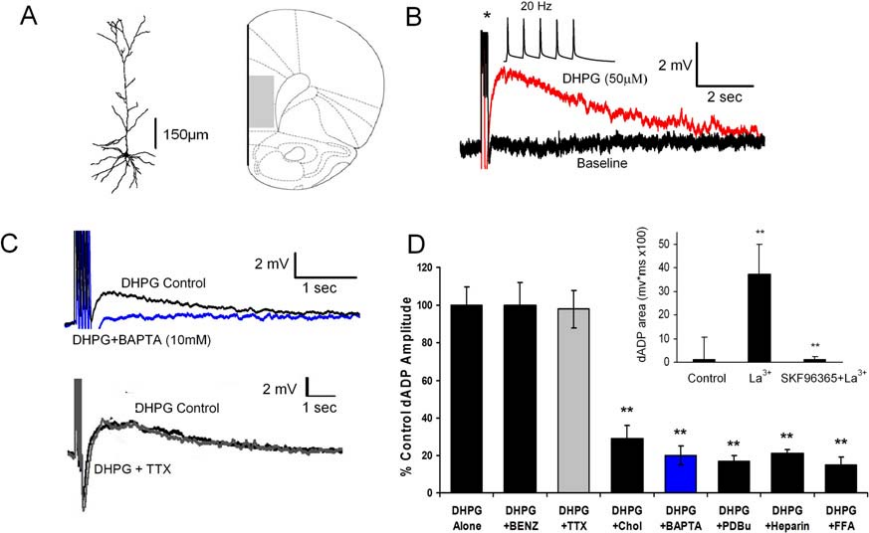
Deep layer 5 pyramidal PFC neurons show a burst-induced nonselective cation current-mediated slow afterdepolarization following activation of group 1 mGluR receptors. (A) Camera lucida reconstruction of a deep layer 5 pyramidal neuron from the PFC (left). (Right) schematic showing the PFC (infralimbic and prelimbic), shaded area where recordings were taken. (B) The group 1 mGluR agonist DHPG (50uM) induced a 20Hz burst-triggered (Inset) dADP (Red) compared to baseline before DHPG application to the bath. (C) (top) Intracellular Ca2+ chelation with BAPTA (10 mM; Blue) for 10 min after establishing whole-cell configuration substantially reduced the dADP induced by a 20 Hz burst and DHPG (50 uM)+BAPTA (10mM) immediately after establishing whole-cell configuration. (bottom) Bath application of the voltage-gated Na^+^ channel blocker, TTX (1 µM, Gray) for 10 min and elimination of action potentials at intensities 10 times the rheobase had no effect on the dADP induced by a 20 Hz burst and DHPG (50 uM)+TTX (1 µM). (D) The effects of the Na+/Ca2+ exchanger blocker benzamil (100 µM); Na+ ion 80% replacement with choline chloride; voltage-gated Na^+^ channel blocker, TTX (1 µM); intracellular Ca2+ chelation with BAPTA (10 mM); PKC activation with PdBU (1 µM),; IP_3_ receptor blockage with heparin (2 mg/ml); and nonselective cation channel blockade with flufenamic acid (100 µM) on the DHPG-(50 µM) and burst-induced dADP. (n>5, **p<0.01) (Inset) The TRPC4/5 potentiator, La^3+^ (100 µM) induced a small burst triggered (5 action potentials @ 20 Hz) dADP in the absence of mGluR activation and was blocked by the broad spectrum nonselective cation channel blocker, SKF96365 (100 µM). (n>5, **p<0.01)

**Figure 11:**
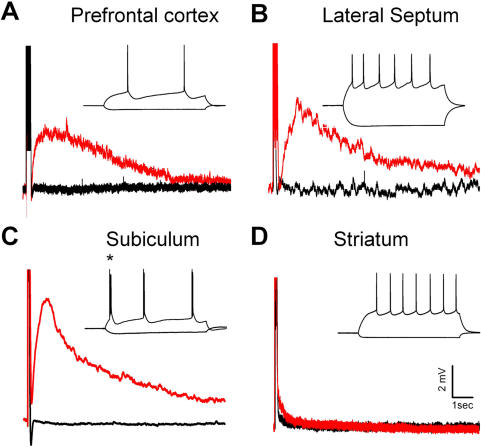
Induction of a nonselective cation current-mediated dADP in brain regions expressing high levels of TRPC mRNA and protein. (A) The group 1 mGluR agonist DHPG (50 uM; Red trace) induces a burst-triggered dADP in the prefrontal cortex (n = 6; p<0.05), (B) Lateral septum (n = 4; p<0.05) and (C) Subiculum (n = 6; p<0.05). Black traces show the baseline before bath application of DHPG (A–D). The inset shows the response to a suprathreshold 600ms square wave input at 200pA or hyperpolarizing input at −100 pA in each cell region (A–D). (D) The lack of a DHPG-induced burst-triggered dADP in medium spiny neurons from the dorsal striatum (n = 6) or nucleus accumbens (n = 4), together labeled Striatum. All recordings were held between −65 and −70mV during the baseline and DHPG application. There were no qualitative differences in the dADP that varied according to the holding potential during the DHPG application in any brain region.

Given the robust expression of these channels in adults and their presence on the surface of the membrane we attempted to identify a function for these channels in the corticolimbic system using whole-cell patch-clamp recording in the deep layer pyramidal neurons. Using the Group 1 metabotropic glutamate receptor (mGluR) agonist DHPG, we observed an action potential burst-induced delayed after-depolarization (dADP) lasting several seconds after the burst. This dADP correlated with the presence of TRPC4 and 5 expression. It was robust in areas such as layer 5 pyramidal neurons in the medial PFC, LS, and SUB where mRNA expression is high, yet it was absent in the nucleus accumbens and striatum where mRNA expression is low (Figure 10A–B, Figure 11A–C). Na^+^ replacement by 80% with choline-Cl substantially reduced the dADP, but did not eliminate it, indicating a substantial Na^+^ ion component to the dADP (Figure 10D). Bath application of the voltage gated Na^+^ channel blocker, tetrodotoxin (1 µM), had no effect on the dADP amplitude, indicating that the current underlying the dADP is not a voltage-gated TTX-sensitive Na^+^ current (Figure 10C–D). Intracellular application of the Ca^2+^ chelator BAPTA (10 mM) significantly reduced the dADP demonstrating the role of intracellular Ca^2+^ in activation (Figure 10C–D). Application of the Na^+^/Ca^2+^ exchanger blocker benzamil (100 µM) failed to reduce the dADP (Figure 10D), ruling out the involvement of a Na^+^/Ca^2+^ exchanger. The dADP was reduced by the protein kinase C activator, PdBU (1 µM), which has also been shown to deactivate TRPC5 channels [41],[42],[43] (Figure 10D). Application of the non-selective cation channel blocker, flufenamic acid (100 µM), and IP_3_ receptor blocker, heparin (2 mg/ml), significantly reduced the dADP (Figure 10D). A small dADP was induced in the absence of DHPG by the trivalent cation La^3+^ (100 µM), which has been shown to potentiate TRPC4 and 5 and block TRPC3, 6, and 7 current in transfected HEK293 cells [9] (Figure 10D inset). This induction of the dADP by La^3+^ was blocked by the non-specific TRP channel blocker, SKF96365 (100 µM ) (Figure 10D inset).

## Discussion

### Expression of TRPC4 and 5 channels in the rodent brain

The TRPC family of nonselective cation channels have been implicated in a number of neuronal processes such as calcium homeostasis, cellular excitability, and axon guidance, yet despite their potential importance, a thorough characterization of these channels in the brain is currently lacking and their exact function and method of activation remains unclear. Determining the regional distribution of these channels in the brain is a fundamental step in understanding their function. In this study, we provide an extensive description of the expression of the TRPC4 and 5 channels, which were found to be the most predominantly expressed TRPC channel mRNAs in the brain. The region-specific mRNA expression of TRPC4 and 5 mRNA throughout the brain was concentrated in areas of the corticolimbic system, such as the PFC, orbitofrontal cortex, LS, HIP, and amygdala. For TRPC4, the highest expression was located in the LS, consistent with previous work by Otsuka et al [17]. We performed detailed quantification of TRPC4 and 5 in the cortical cell layers and found a uniform distribution of TRPC4 through layers 2–6 with no labeling in layer 1, while TRPC5 mRNA showed dense labeling specifically in layers 2–3 and 5–6. These results suggest that TRPC5 mRNA is localized to pyramidal neurons found in high density in these two regions of the PFC although further work is needed to demonstrate this directly. The uniform distribution of TRPC4 in the cortical layers suggests it is distributed throughout many cell types. Expression of both TRPC4 and 5 in the cell body layer of the HIP provides evidence that these channels are present in pyramidal neurons in the HIP. These results are consistent with previous immunohistochemical work by Chung et al. showing high expression of TRPC5 in the cell body layer of the HIP [22]. The striking absence of either mRNA or protein in the stratum radiatum and stratum lacunosum moleculare suggests a lack of TRPC4 or 5 expression in the interneurons in these regions, although this would best be examined using immunohistochemistry.

### TRPC5 channel transport along neuronal projections

The TRPC channels have been implicated in both presynaptic and postsynaptic neuronal processes, however, the synapse specific expression and function of these channels is currently unknown. In our studies we observe a disparity in the mRNA and protein levels of TRPC5 in some brain regions, suggesting that the protein may be transported along neuronal projections into the presynaptic terminals, thus providing evidence for a presynaptic function of the channel. The protein expression of TRPC4 and TRPC5 correspond with the mRNA expression for each channel in regions such as the PFC, LS, and HIP; however, in the nucleus accumbens TRPC5 mRNA is expressed at low to moderate levels yet the protein is expressed at moderate to high levels. Also, previous studies by von Bohlen et al. (2006) show TRPC5 protein expression in the substantia nigra using immunohistochemistry, however, our studies show a lack of TRPC5 mRNA expression in this region [28]. It is not uncommon that mRNA and protein levels do not match [29]. These differences can be attributed to regulation at the transcriptional or translational level, differences in half-lives of the mRNA and protein due to differences in rates of synthesis and degradation, or mRNA and protein transport and trafficking [24]. However, a more likely explanation for our results in the nucleus accumbens is a differential pre vs. postsynaptic expression of the TRPC5 mRNA and protein. For example, the deep layer pyramidal neurons in the PFC send a substantial projection to the nucleus accumbens [30]–[32], therefore it is possible that TRPC5 mRNA is transcribed and translated in the cell bodies located in the PFC and the protein is subsequently transported into presynaptic terminals located in the accumbens. Future studies examining the sub cellular localization of TRPC5 protein may help elucidate whether the channel is expressed in the presynaptic or postsynaptic terminals or in the dendrites.

### TRPC channel regulation of neuronal growth and plasticity

The transport of the TRPC5 channels into the presynaptic terminals and the up-regulation of TRPC4 and 5 protein levels in the HIP of P48 compared to P0 mice suggests that these channels may be involved in neuronal development. TRPC5 is thought to be involved in regulating neuronal growth in HIP primary culture cells [15],[10]. Stimulation of cultured E18 HIP neurons by growth factors invokes a negative feedback mechanism involving the vesicular translocation and activation of TRPC5 channels in the membrane of neuronal growth cones leading to the cessation of growth [10]. This work in cell culture systems is consistent with our finding of increased developmental expression of TRPC5 in HIP, PFC, SUB, and entorhinal cortex. It's intriguing, though speculative, that TRPC5 may serve as a switch to terminate neuronal growth as the animal reaches maturity and neural circuitry becomes fully developed.

Since growth factors modulate synaptic plasticity in the adult mouse HIP [33]–[36], TRPC5 activation by growth factors may be important for synaptic plasticity. It's interesting there is a fraction of TRPC5 that remains intracellular at both P0 and P48. It's possible that TRPC channel trafficking to the surface may be subject to other signals, such as Gq-coupled receptor/PLC cascades that have been shown to activate TRPC channels and modulate neuronal plasticity in the HIP and cortex [37]–[39].

### TRPC channel modulation of neuronal excitability in the corticolimbic system

The dense pyramidal neuron-specific corticolimbic expression of TRPC4 and 5 channels in the brain suggests that they may be involved in modulating synaptic plasticity associated with learning and memory. TRPC 4 and 5 channels are activated in vitro by mGluR receptor stimulation, which are receptors that have been implicated in neuronal plasticity in the HIP [5], [38] [40] [41]. In brain regions expressing high levels of TRPC4 and 5 mRNA and protein we stimulated Gq-coupled receptors using the group 1 mGluR agonist DHPG to induce a dADP that is triggered by Ca^2+^ entry coming from a brief burst of 5 action potentials. Action potential bursting is a neuronal output mode thought to be relevant for routing information and signaling novel and salient events [42],[43]. Recent work by our laboratory indicates that the DHPG-induced burst-triggered dADP in the PFC is mediated specifically by mGluR5 receptors (Sidiropolou, submitted). Furthermore, we report that the dADP is mediated by a nonselective cation channel current that is dependent on intracellular Ca^2+^ and IP_3_ receptor function, which suggests that the dADP is activated through a G_q_/PLC coupled signaling pathway. The blockade of the sADP by the TRPC channel blocker, SKF96395 and the ability of the TRPC4 and 5 potentiator, La ^3+^ to induce the sADP in the absence of any group 1 mGluR simulation provides evidence for TRPC4 and 5 involvement in mediating the sADP. Furthermore, we observed a substantial reduction in the dADP amplitude in response to PKC activation which is consistent with the report that in HEK cells, TRPC5 channels are desensitized by PKC phosphorylation of T972 located in the C-terminal region [44],[45],[46].

Further support for the hypothesis that the dADP is mediated by TRPC4 or 5 channels comes from patch-clamp recordings in brain regions where TRPC4 or 5 mRNA was not detected. For example, the dADP is completely absent in striatal medium spiny neurons where TRPC4 and 5 mRNA is not present, yet this is not due to the lack of mGluR expression in the medium spiny neurons[47], [48]. In fact, mGluR1 and 5 receptors are expressed and their activation induces long term depression in this area [48],[49]. However, although we have presented a case for TRPC4 or 5 involvement in the mGluR-induced dADP, our results remain correlative and future studies dedicated to establishing a causal relationship are necessary. If in fact mGluR stimulation activates TRPC4 or 5 channels producing a dADP, then in addition to the proposed presynaptic role this would provide a postsynaptic function for these channels in the corticolimbic system.

## Materials and Methods

### Micro dissections of brain regions

For all brain regions, slices were taken from male rats and mice (8–10 weeks old for all experiments with the exception of the developmental and surface expression experiments where the ages are specified). Animals were anesthetized by halothane inhalation and their brains quickly removed and placed into ice-cold artificial cerebral spinal fluid (ACSF). The brain was mounted and coronal slices (500 µm for rat, 250 µm for mice) were cut using a vibratome. Each region was dissected while the slices were in ice chilled ACSF and immediately frozen on dry ice upon extraction.

### RNA extraction and reverse transcription

Total RNA was isolated using Trizol reagent (Invitrogen, Carlsbad, CA, USA) in accordance with the protocol. The RNA was resuspended in DEPC-treated H_2_O and DNase-treated to eliminate any DNA contamination using DNA-free–(Ambion, Austin, TX, USA). The concentration of the RNA was determined by UV absorbance using a Nanodrop spectrophotometer. The RNA was reverse transcribed with random hexamer primers (Invitrogen, Carlsbad, CA, USA) using Superscript II reverse transcriptase (Invitrogen, Carlsbad, CA, USA). The cDNA was purified on a spin purification column (Qiagen, Valencia, CA, USA) and the concentration was determined by UV absorbance using a Nanodrop spectrophotometer.

### Quantitative real-time PCR

The real-time primers were designed as 23–25 oligonucleotide sequences that amplify a 100–150 base pair gene product. Primers were checked against all known rat gene sequences to ensure specificity and were designed to maximize detection of all known splice variants of the TRPC genes. The real-time PCR reaction was optimized by running a standard curve for each primer set in dilutions of whole brain rat cDNA (1, 0.5, 0.25, 0.125, and 0.0625), with 100 ng of cDNA in the 1× concentration. From this standard curve, the efficiency for each gene was determined using the slope and R^2^ value according to the method proposed by Pfaffl [50]. The reactions were carried out using SYBR Green PCR Master Mix (Ambion, Austin, TX, USA) in a Stratagene MXP3000 real-time PCR thermal cycler (Stratagene, La Jolla, CA, USA). The amplification plots, melting curves, and standard curve were assessed for primer quality and the product was run on a gel to ensure amplification of a single product of the correct size. The TRPC channel expression was assessed in the different brain regions by running real-time PCR reactions of 100 ng of the cDNA transcribed from total RNA isolated from the dissections. In all reactions GAPDH was used as a positive control to which the results were normalized.

For the relative TRPC channel expression analysis, the cycle threshold values for the reactions were normalized to GAPDH and then expressed as a percentage of 100 relative to each other. For the region-specific TRPC channel expression, the cycle threshold values for the reactions were normalized to GAPDH and expressed as a fold increase relative to expression levels in whole rodent brain. This analysis emphasizes that TRPC4 and 5 have specific expression in those regions compared to levels in whole brain and are not expressed at equal levels throughout the brain.

### Molecular cloning of TRPC4 and 5

The TRPC4 and 5 channels were cloned from cDNA reverse-transcribed from total RNA isolated from adult rat HIP tissue using Trizol (Invitrogen, Carlsbad, CA, USA). Primers to each of the channels were designed to the 3′ and 5′ ends of the mRNA sequences. The 5′primer for TRPC4 contained a XhoI site and the 3′primer contained a BamHI site for cloning into pBluescript (Stratagene, La Jolla, CA, USA). The 5′primer for TRPC5 contained a XhoI site and the 3′primer contained a XbaI site for cloning. The TRPC4 and 5 sequences were amplified using Long Template DNA polymerase (Roche, Indianapolis, IN) and the products were cloned into pBluescript and confirmed by sequencing. The TRPC5 sequence was cloned into pcDNA-FLAG as a fusion protein with a FLAG tag on the C-terminal end. The clone was confirmed by sequencing.

### In situ hybridization of TRPC4 and 5

The in situ hybridization experiments were carried out according to the methods described by Gold and Zachariou [51]. Slide-mounted, fresh-frozen tissue sections were used. Halothane-anesthetized animals were decapitated and their brains removed and rapidly frozen on dry ice. Coronal, fresh-frozen sections were cut at 14 µm in a cryostat, thaw-mounted onto Superfrost Plus (Fisher Scientific, Pittsburgh, PA, USA) glass slides, and stored at −80°C until use. The sections were fixed in 4% paraformaldehyde, and washed in PBS, PBS+glycine, and 0.25% acetic anhydride in 0.1M triethanolamine. The sections were then dehydrated and delipidated in 50%, 75%, 95%, and 100% EtOH and 100% chloroform. Sections were hybridized for 18 hours at 60°C in hybridization buffer containing deionized formamide (40%), dextran sulfate (10%), 1× Denhardt's solution, 4×SSC, denatured and sheared salmon sperm DNA (1 mg/mL), yeast tRNA (1 mg/mL), dithiothreitol (10 mM), and either S^35^-labeled RNA probe (S^35^, Perkin Elmer, Waltham, MA, USA) or an identical, unlabeled RNA probe as a control. The probes used were a 188 bp S^35^-labeled probe for TRPC4 and a 300 bp S^35^-labeled probe for TRPC5 which were transcribed using T3 polymerase (Ambion, Austin, TX, USA) from the TRPC4 and 5 pBluescript clones described above and purified on a spin purification column. The counts per million (cpm) of the probes were assessed using a scintillation counter and added to the hybridization buffer at a concentration of 10×10^6^ cpm/ml. After hybridization, the sections were washed in sodium citrate buffer (SSC)+sodium thiosulfate and placed on film for 3–7 days before development.

### Quantification of TRPC4 and 5 expression

The expression of TRPC4 and 5 from the in situ hybridization film in the cortex layers was quantified by measuring the signal intensity of a 125 µm^2^ region of interest being quantified. The signal intensity of each region was normalized to an identically sized background region, where no tissue was present. The rationale for using the background region as the control is that because we are examining the expression of TRPC4 and 5 in different brain regions within the same slice, instead of the differences in expression in the same region across different slices, using a region of the tissue without a specific signal has an advantage because it does not assume expression or lack of expression in any brain regions. The controls show a homogenous background level.

For the qualitative assessment of the TRPC4 and 5 signal from the in situ hybridization film, the signal intensity in various brain regions was scored by three independent investigators according to the scale of 0<+<++<+++. The average intensity for each brain region was recorded.

### Expression of TRPC5 in HEK293 cells

One day before transfection, 0.5–2×10^5^ HEK293 cells in 1 ml of Opti-MEM Reduced Serum Medium (Invitrogen, Carlsbad, CA, USA) without antibiotics were plated into 6-well plates so that the cells would be 90–95% confluent at the time of transfection. One µg of TRPC5-Flag plasmid DNA (pTRPC5-F) or control pGFP DNA was diluted into 50 µl of Opti-MEM Reduced Serum Medium (Invitrogen, Carlsbad, CA, USA). Lipofectamine 2000 (Invitrogen, Carlsbad, CA, USA) in a ratio of 1:3 (plasmid: reagent) was diluted into 50 ul Opti-MEM I Medium (Invitrogen, Carlsbad, CA, USA) and incubated for 5 min at RT. The Lipofectamine and DNA dilutions were combined and incubated at room temperature for 20 min, then added to the plates containing the HEK293 cells. The cells were incubated at 37°C with 95% O_2_ and 5% CO_2_. To identify the optimal transfection conditions, a time course of 18, 24, 36, and 48 hours post-transfection harvesting was performed. A mock transfection control of empty vector and a nontransfected control were included.

### Cross-linking Procedure

C57/Bl6 P0 and P48 day old-mice were rapidly decapitated and their brains were extracted and placed into ice-cold ACSF. The whole hippocampus was dissected from 300 µm slices cut on a vibratome (Leica, Nussloch, Germany) and placed into tubes containing ice-cold ACSF on ice. The slices were cross-linked by the method described by Boudreau et al [27]. The slices were treated with 2 mM BS^3^ (in 5mM NaCitrate buffer, pH 5.0; Pierce, Rockford, IL, USA) for 30 minutes at 4°C with shaking. The reaction was quenched by the addition of 100 mM glycine for 10 minutes at 4°C with shaking. Control slices that did not receive BS^3^ were also incubated for 30 minutes at 4°C with shaking and received 100 mM glycine for 10 minutes at 4°C with shaking. The tubes were spun down, the supernatant was aspirated, and the cells were immediately lysed by sonicating in boiling 1% sodium dodecyl sulphate (SDS) lysis buffer. The samples were boiled for 10 minutes. The protein concentration was determined using the BCA assay (Pierce, Rockford, IL, USA) and 20 µg of protein was loaded onto a 5–16% gradient sodium dodecyl sulfate–polyacrylamide gel electroporesis (SDS-PAGE) gel. The protein was transferred from the gel to Hybond ECL nitrocellulose membrane (Amersham, Piscataway, NJ, USA) for immunoblotting.

### Immunoblot Procedure

Homogenates were obtained by sonicating tissue suspensions in boiling 1% SDS lysis buffer. Protein concentrations were determined by the BCA assay (Pierce, Rockford, IL, USA). Samples are loaded onto 10% SDS–PAGE gels (100 µg protein/lane). Preliminary experiments were performed to demonstrate that these amounts are in the linear range as determined by densitometric analysis. Gels were transferred to Hybond ECL nitrocellulose membrane (Amersham, Piscataway, NJ, USA) for immunoblotting. The membranes were blocked in 5% milk for 1 hour, then primary antibody (TRPC4, 1∶1000, gift from Dr. Bonanno; TRPC5, 1∶400, Alomone, Jersusalem, Israel or Sigma-Aldrich, St. Louis, MO, USA; β-actin monoclonal, 1∶1000, Sigma-Aldrich, St. Louis, MO, USA) for either 1 hour at room temperature or overnight at 4°C with shaking. The membranes were incubated in the appropriate secondary antibody (GαRb, 1∶8000 or GαM, 1∶8000, Pierce, Rockford, IL, USA) for 1 hour at room temperature with shaking and developed with enzymatic chemiluminescence (ECL, Amersham Biosciences, Piscataway, NJ, USA) reagent.

### Patch Clamp Procedures

The procedures used were similar to our previous study of PFC pyramidal neurons [52]–[54]. To ensure reliable recordings from healthy cells, we implemented several criteria for choosing a cell which include: ability to visualize the cell; resting potential more negative than −60 mV; series resistance less than 30 MΩ. To ensure recording exclusively from pyramidal neurons, we include biocytin in the pipetted for post-hoc verification of pyramidal neuronal morphology. We will use histological methods to determine the neuron type.

#### Slice Preparation

For all brain regions slices were taken from mature rats (5–8 weeks old). Halothane-anesthetized rats were perfused through the heart with chilled ACSF and the brain. The slices (300 µm) were cut using a Vibratome 3000 (Leica, Nussloch, Germany). Slices were incubated for 20–40 min in warm (34–35°C) ACSF, then held at RT until they were moved to the recording chamber. For recording, slices were transferred to a chamber on a fixed stage of an Olympus BX51WI equipped with differential interference contrast optics. Recordings were obtained under visual control using Dage-MTI (Michigan City, IN) tube camera. All experiments were performed during continuous perfusion with ACSF at 32–35°C.

#### Solutions and Drugs

Recording ACSF consists of (in mM): 125 NaCl, 25 glucose, 25 NaHCO_3_, 2.5 KCl, 1.25 NaH_2_PO_4_, 2 CaCl_2_, and 1 MgCl_2_, pH 7.4 (bubbled with 95% O_2_ and 5% CO_2_). Kynurenic acid (2.5 mM), SR 95531 (4 µM), and atropine (1 µM) was added to the ACSF to eliminate glutamate (NMDA and AMPA), GABA_A_ and muscarinic acetylcholinergic synaptic activity, respectively if necessary. Internal solution compositions are described in Patch-clamp recordings below.

#### Current-Clamp Recordings

Whole-cell, current-clamp recordings were made from the soma using a BVC-700 amplifier (Dagan, Minneapolis, MN). Patch-clamp electrodes was fabricated from thick-walled borosilicate glass and fire polished to resistances of 3–4 MΩ in the bath. The intracellular solution for whole-cell current-clamp recordings contains (in mM): 115 K-gluconate, 20 KCl, 10 Na_2_-phosphocreatine, 10 HEPES, 2 Mg-ATP, and 0.3 Na-GTP, pH 7.3, and 0.1% biocytin (for subsequent morphological identification). Data were stored on a PC Pentium IV via an ITC-18 interface (Instrutech, Port Washington, NY). Data acquisition and analysis is performed using custom software (DataPro 5.0, N. Spruston and D. Cooper) running under Igor Pro (WaveMetrics, Lake Oswego, OR). Voltage was digitized at 20 kHz and filtered at 5 kHz.

#### Histological Procedures

After recording, the slices were placed in paraformaldehyde (4%) and refrigerated at 4°C for 2 weeks. We processed the biocytin-filled cells using an avidin–horseradish peroxidase reaction using the Vectastain ABC Kit (Vector Labs, Burlingame,CA). Processed slices were mounted on microscope slides using Mowiol. Neuron reconstructions was performed using a Neurolucida system (MicroBrightField, Inc., Williston, VT) and a Olympus BX51 with a 100× oil-immersion objective.

## Supporting Information

Figure S1 Expression of β-actin protein under cross-linking conditions. (A) Quantification and representative bands (top inset) of β-actin protein levels in cross-linked and non cross-linked P0 mouse hippocampal samples (n = 6, p = 0.42) (B) Quantification and representative bands (top inset) of beta actin protein levels in cross-linked and non cross-linked P48 mouse hippocampal samples (n = 6, p = 0.43)
(0.89 MB TIF)
