# Supplementary figures and images for: Corticolimbic Expression of TRPC4 and TRPC5 Channels in the Rodent Brain
Source: PLoS One. 2007 Jun 27;2(6):e573. doi: 10.1371/journal.pone.0000573 (PMC1892805; doi:10.1371/journal.pone.0000573)

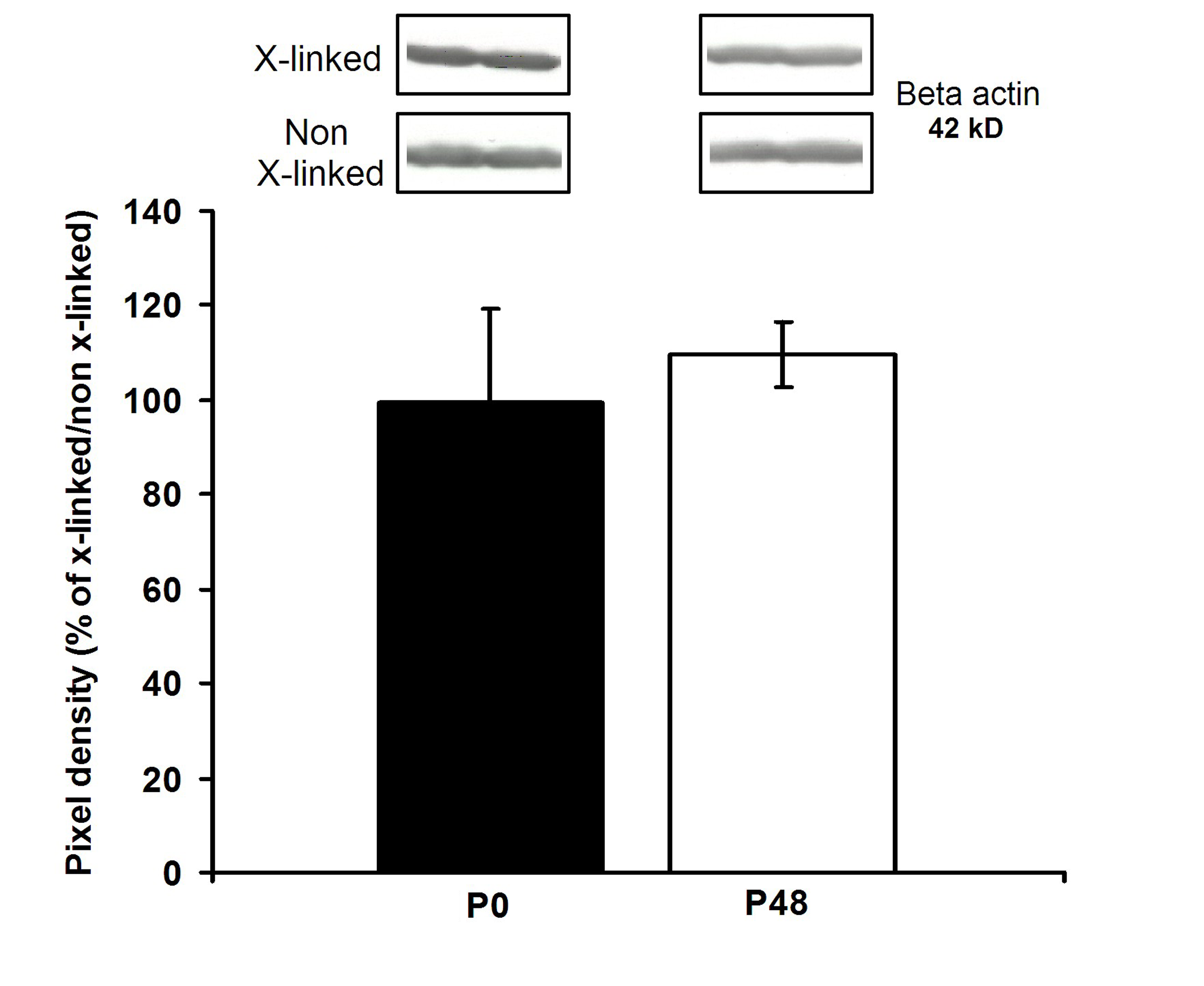

Supplement: Figure S1 — Expression of β-actin protein under cross-linking conditions. (A) Quantification and representative bands (top inset) of β-actin protein levels in cross-linked and non cross-linked P0 mouse hippocampal samples (n = 6, p = 0.42) (B) Quantification and representative bands (top inset) of beta actin protein levels in cross-linked and non cross-linked P48 mouse hippocampal samples (n = 6, p = 0.43) (0.89 MB TIF) [file pone.0000573.s001.tif]
